# Rare Case of Central Pontine Myelinolysis: Etiological Dilemma

**DOI:** 10.7759/cureus.19644

**Published:** 2021-11-16

**Authors:** Mohan V. Sumedha Maturu, Aravind Varma Datla, Vinayagamani Selvadasan, Sibasankar Dalai

**Affiliations:** 1 Neurology, Medicover Hospitals, Visakhapatnam, IND; 2 Internal Medicine, Medicover Hospitals, Visakhapatnam, IND; 3 Neuro & Interventional Radiology, Vadamalayan Hospital, Madurai, IND; 4 Interventional Neuroradiology, Medicover Hospitals, Visakhapatnam, IND

**Keywords:** central pontine myelinolysis, anti-ssa antibody, brain magnetic resonance, distal renal tubular acidosis, hypokalaemia, sjogren's

## Abstract

Central nervous system (CNS) involvement in Sjogren's syndrome (SS) has a broad spectrum of presentations. We present a 33-year-old with sudden onset, rapidly progressive quadriplegia, severe dysarthria, bilateral facial palsy, bulbar palsy, and hypernatremia. The MRI of the brain revealed hyperintensity in the central pons diffusion-weighted imaging, T2-weighted imaging, and fluid-attenuated inversion recovery (FLAIR) without abnormal contrast enhancement, consistent with central pontine myelinolysis. However, there was no antecedent history of hyponatremia with rapid correction. The patient responded excellently to sodium correction and pulse methylprednisolone therapy and was erroneously diagnosed as idiopathic hypernatremic osmotic demyelination. One year later, she presented with vague constitutional symptoms, renal tubular acidosis type-1 (distal), hypokalemia with associated myopathy. Subsequent testing for anti-Sjögren's-syndrome-related antigen A (SSA)/Ro autoantibodies and a biopsy of the minor salivary gland established the diagnosis of primary Sjogren syndrome (pSS). Remission was achieved with oral prednisolone after her discharge. Neurological signs can be the initial presentation that precedes the classical systemic manifestations of multisystem autoimmune disorders like pSS. In the event of osmotic demyelination, when antecedent hyponatremia with rapid correction is not there, we suggest evaluating for possible autoimmune etiology.

## Introduction

Sjögren’s syndrome (SS) is a chronic, slowly progressing autoimmune disease characterized by lymphocytic infiltration of the exocrine glands resulting in xerostomia and dry eyes (keratoconjunctivitis sicca) [1]. The prevalence of primary Sjögren’s syndrome (pSS) is between 0.5 and 1% (second most common rheumatological disorder after rheumatoid arthritis), while 5-20% of patients with other autoimmune diseases suffer from SS (secondary) [1,2]. Middle-aged women are primarily affected (female-to-male ratio, 9:1). Onset usually occurs in the fourth or fifth decade of life, although SS may occur at any age, including childhood [1,3]. Extraglandular (systemic) manifestations are seen in one-third of patients with SS, mainly affecting the joints, skin, lungs, kidneys, liver, lymphatic tissue, and peripheral nervous system (PNS) [4,5]. There are numerous diagnostic criteria that can establish the diagnosis of pSS [3,6].

The peripheral nervous system (PNS) manifestations of pSS are well established. Although central nervous system (CNS) involvement is recognized, its pathogenesis and characteristics are varied and poorly understood.

Alexander et al., in 1981, first described CNS involvement in a series of eight patients and suggested a direct etiopathogenetic role of the anti-Ro [anti-Sjögren's-syndrome-related antigen A (SSA)] antibodies [7]. A review of the literature revealed 88 cases of CNS involvement in pSS in the form of case reports or series. In some patients, the CNS involvement may precede clinical diagnosis by multiple years and may lead to an underestimation of other neurological and systemic diseases [8]. This case report highlights the rare occurrence of CNS involvement as the initial manifestation of pSS.

## Case presentation

**Figure 1:**
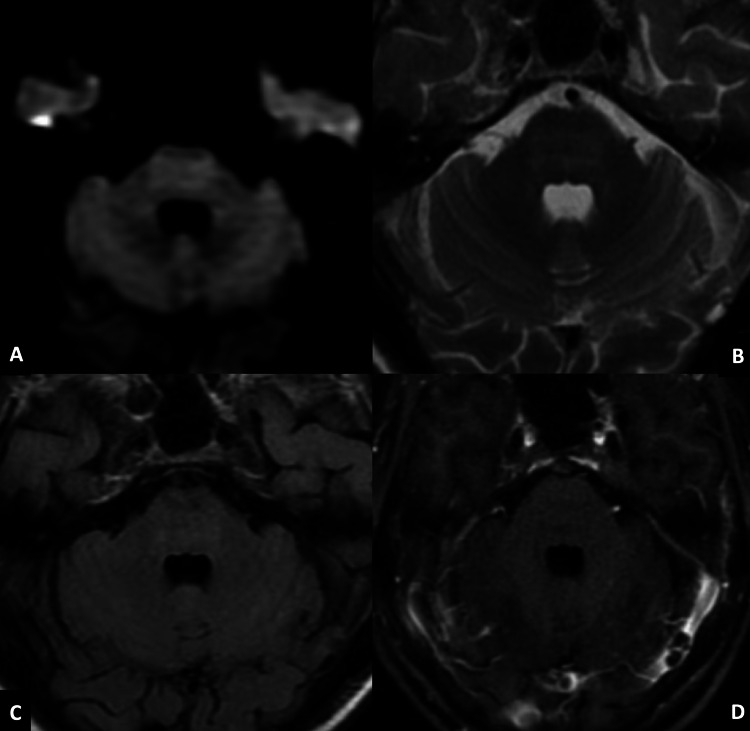
Brain MRI of the patient. Hyperintense signal in central pons with peripheral sparing in axial diffusion-weighted image (A), axial T2-weighted image (B), and axial FLAIR (C). Post-contrast axial T1-weighted image (D) does not show any abnormal enhancement. FLAIR: fluid-attenuated inversion recovery.

A 33-year-old female with no prior medical comorbidities, who recently gave birth to a healthy girl child four months ago, was brought to the emergency department with sudden onset weakness of both upper and lower limbs that started four days prior and rapidly progressed to a state of quadriplegia. She was conscious and obeyed simple commands with eyes and mouth; however, she had severe dysarthria. She had bilateral facial palsy and bulbar palsy. She had flaccid, hyporeflexic, pure motor quadriplegia with limbs showing only a subtle withdrawal flicker to pain. MRI of the brain revealed hyperintensity in the central pons in diffusion-weighted images (Figure 1A), T2-weighted images (Figure 1B), and fluid-attenuated inversion recovery (FLAIR) images (Figure 1C) without abnormal contrast enhancement (Figure 1D), consistent with central pontine myelinolysis (CPM) (Figure 1).

The biochemical analysis showed hypernatremia while the remaining electrolytes were normal. The rest of the blood workup was unremarkable. Relatives denied an antecedent history of hyponatremia with rapid correction. The patient was started on sodium correction and was given five days intravenous (IV) pulse methylprednisolone 1 g/day to stabilize the blood-brain barrier. The patient recovered significantly to normal power. She was then considered to have idiopathic hypernatremic osmotic demyelination and was discharged with a modified Rankin Scale score (mRS) of 0. 

One year later, she presented to the neurology department with a one-week history of generalized fatigue, diffuse myalgias, and three days history of rapidly progressive weakness of all four limbs making her wheelchair-bound one day before the presentation. Her initial vital signs were unremarkable. She was noted to have a pure motor flaccid symmetric quadriparesis with proximal more than distal weakness and generalized hyporeflexia. Clinical examination of other systems was normal. Nerve conduction studies (NCS) done on day three of onset of weakness demonstrated reduced compound muscle action potential (CMAP) amplitudes of bilateral tibial and peroneal nerves with absent F waves and H reflexes. CMAP of tested nerves in upper limbs showed preserved amplitudes with normal distal latency and absent F waves. There were no conduction blocks. The sensory conduction study of all the tested nerves in all four limbs was normal. Cerebrospinal fluid (CSF) analysis did not show albumin-cytological dissociation. Therefore, acute motor axonal neuropathy (AMAN) variant of Guillain-Barré syndrome (GBS) or hypokalemia-related electrophysiological abnormalities were considered. A basic metabolic panel revealed severe hypokalemia (potassium 2.2 mEq/L). Arterial blood gas (ABG) and 24-hour urine analysis showed metabolic acidosis, consistent with renal tubular acidosis type-1 (distal). Autoimmune workup was positive anti-SSA/Ro autoantibodies. The biopsy of the minor salivary gland was pathognomonic. The patient was diagnosed with pSS according to the new classification criteria proposed by the American College of Rheumatology (ACR) and the European League Against Rheumatism (EULAR). Overall clinical, electrical, and biochemical data suggest the presence of renal tubular acidosis with secondary hypokalemia-related quadriparesis due to pSS.

The patient was given intravenous (IV) potassium supplementation through a peripheral vein at a rate not exceeding 10 mEq/hour and subsequently was shifted to oral liquid formulation in the form of a syrup. Oral sodium bicarbonate supplementation was given at a dose of 1 mEq/kg/day for renal tubular acidosis. With potassium correction, there was a rapid recovery in the power of all four limbs within 24 hours of admission. The patient was initiated on 1 mg/kg/day of oral prednisolone and was referred to a rheumatologist for further management. She remained asymptomatic on her six-month follow-up.

## Discussion

This case report describes SS with CNS involvement (CNS-SS), misdiagnosed as idiopathic CPM. Considering that there was no prior history of hyponatremia with rapid correction, the dramatic response to IV steroids should have raised suspicions about a possible autoimmune pathology. The patient's subsequent presentation with correlating clinical, laboratory, and electrophysiological findings with positive anti-SSA confirmed the diagnosis of SS. Based on the current knowledge, a retrospective look into her initial presentation revealed that the lesion could have been the initial CNS manifestation of pSS.

The frequency of neurological manifestations of pSS is challenging to assess due to the lack of broad population studies, but it is estimated to be 20% [5,9]. CNS involvement is less frequent, and the reported manifestations vary (Table 1).

**Table 1:** Neurological manifestations of primary Sjogren syndrome.

Peripheral nervous system involvement	Central nervous system involvement
Axonal polyneuropathies: (i) symmetric pure sensory peripheral neuropathy, (ii) symmetric sensorimotor peripheral neuropathy	Focal manifestations (for example, motor/sensory deficit)
Sensory ganglioneuronopathy	Aseptic meningoencephalitis
Motor neuropathy	Myelopathy
Small-fibre neuropathy	Headache
Multiple mononeuritis	Cognitive disorders
Trigeminal and other cranial nerves neuropathies	Mood disorders
Autonomic neuropathies	Seizures
Demyelinating polyradiculoneuropathy	Pyramidal signs
	Brainstem signs
	Cerebellar syndrome
	Encephalopathy
	Spinal cord involvement
	Multiple sclerosis-like disease

Around 5% of the pSS patients develop CNS features and may precede the onset of sicca symptoms [8,10]. The ever-changing classification criteria and referral bias might account for the varying estimates of the prevalence of pSS and its CNS manifestations.

Immune-mediated mechanisms may play a role in CNS disease in SS. Alexander et al. found lymphocytosis, raised immunoglobulin G (IgG) index, and oligoclonal bands on electrophoresis during the CSF examination of SS patients with active CNS disease, suggesting intrathecal migration of lymphocytes and antibody synthesis [11]. Sanders et al. observed intrathecal complement activation on examining CSF from CNS-SS patients [12]. Histopathology of brain tissue in some CNS-SS patients demonstrated a small vessel mononuclear inflammatory and ischaemic/haemorrhagic vasculopathy. Both Ro+ and Ro− patients with SS can develop CNS disease. However, there is an association between anti-Ro positivity and the severity of CNS disease. Anti-Ro positivity in a known SS patient presenting with CNS manifestations has prognostic value [13].

MRI has a sensitivity of 75-88% in patients with CNS-SS. The majority of the lesions appear as hyperintense signals in the subcortical and periventricular white matter [14]. Other neurodiagnostic modalities such as CSF analysis, computed tomography (CT) scan, electroencephalogram, and cerebral angiography are less revealing [3]. CPM as the initial manifestation of CNS-SS is exceedingly rare. Two such cases have been reported previously by Nagashima et al. in 1996 and Yoon et al. in 2000 [15,16].

Neurological deterioration in CPM often progresses for about one week and then stabilises or improves. Spastic quadriplegia, locked-in syndrome, and dysarthria are also noticed. The size of the lesion is not analogous to the clinical severity. However, the size of the lesion may decrease with clinical improvement, but larger lesions often persist [16].

The treatment approach is conservative and mainly depends on the disease severity. Our patient responded to the pulse therapy with methylprednisolone and is in remission with 1 mg/kg oral prednisolone. Acute attacks can be managed by pulse steroids, pulse cyclophosphamide, intravenous immunoglobulins (IVIG), or plasmapheresis [16]. Remission can be maintained with cyclophosphamide, azathioprine, or mycophenolate mofetil. Due to their pathogenetic role, monoclonal antibodies targeting B-cells (including rituximab and belimumab) are increasingly used [17]. However, the results are variable. A deeper understanding of disease mechanisms and the establishment of prognostic markers with more tailored treatment regimens are necessary for obtaining the best possible outcomes in such individuals.

## Conclusions

In the event of osmotic demyelination, when antecedent hyponatremia with rapid correction is not there, we suggest evaluating for possible autoimmune etiology, giving a trail of IV pulse steroids, and keeping the patient under close clinical follow-up to document the evolution of the syndrome. Neurological signs can be the initial presentation that precedes the classical systemic manifestations of multisystem autoimmune disorders like pSS.
